# Correction: *Prevalence of and factors associated with adopting bone health promoting behaviours among people with osteoporosis in Taiwan: a cross-sectional study*

**DOI:** 10.1136/bmjopen-2017-015980corr1

**Published:** 2018-01-24

Chen P, Lin M, Huang T, *et al*. Prevalence of and factors associated with adopting bone health promoting behaviours among people with osteoporosis in Taiwan: a cross-sectional study. *BMJ Open* 2017;7:e015980. doi: 10.1136/bmjopen-2017-015980

The Funding statement should read:

**Funding** The study was supported by a grant from the Taiwan Formosa Plastic Company (FCRPF690011) and Chang Gung Memorial Hospital (BMRP148) as well as (CMRPGMF0012).

The Ethics approval statement should read:

**Ethics approval** This study was approved by the institutional review board of the

ethical committee of Chang Gung Memorial Hospital (IRB 104-9925C).

